# The Role of L-type Calcium Channels in Olfactory Learning and Its Modulation by Norepinephrine

**DOI:** 10.3389/fncel.2017.00394

**Published:** 2017-12-11

**Authors:** Abhinaba Ghosh, Samantha J. Carew, Xihua Chen, Qi Yuan

**Affiliations:** Laboratory of Neuroscience, Department of Biomedical Sciences, Faculty of Medicine, Memorial University of Newfoundland, St. John’s, NL, Canada

**Keywords:** olfactory learning, memory, L-type calcium channel, norepinephrine, NMDAR

## Abstract

L type calcium channels (LTCCs) are prevalent in different systems and hold immense importance for maintaining/performing selective functions. In the nervous system, CaV_1.2_ and CaV_1.3_ are emerging as critical modulators of neuronal functions. Although the general role of these calcium channels in modulating synaptic plasticity and memory has been explored, their role in olfactory learning is not well understood. In this review article we first discuss the role of LTCCs in olfactory learning especially focusing on early odor preference learning in neonate rodents, presenting evidence that while NMDARs initiate stimulus-specific learning, LTCCs promote protein-synthesis dependent long-term memory (LTM). Norepinephrine (NE) release from the locus coeruleus (LC) is essential for early olfactory learning, thus noradrenergic modulation of LTCC function and its implication in olfactory learning is discussed here. We then address the differential roles of LTCCs in adult learning and learning in aged animals.

## Introduction

L-type calcium channels (LTCCs), characterized by their long-lasting activity and sensitivity to dihydropyridine, are members of the high voltage gated calcium channel family. Like other calcium channels of this type, they are composed of multiple subunits including α1, the pore-forming subunit containing the voltage sensor, which dictates the nomenclature of the channel subtype. Between CaV_1.1–1.4_, CaV_1.2_ and CaV_1.3_ are most commonly found in the nervous system. CaV_1.3_ has a lower activation voltage compared to CaV_1.2_ (Hofmann et al., 2014). LTCCs function to facilitate coupling, mainly excitation-contraction, excitation-secretion and excitation-transcription; the latter being crucial for neuronal function and memory formation including olfactory memory (Jerome et al., 2012; Berger and Bartsch, 2014; Ghosh et al., 2017).

In this review article, we present evidence of the functional roles of LTCCs in synaptic plasticity and learning, focusing on olfactory learning as a model system. The role of norepinephrine (NE) via β-adrenoceptors (βARs) in modulating LTCCs and early odor preference learning are detailed. Finally, the differential roles of LTCCs in learning during development and aging are discussed.

## LTCCs in Synaptic Plasticity and Learning

LTCC dependent calcium entry induces varied downstream molecular cascades, the functions of which are vital to understanding the importance of the channels. The C-terminal of the LTCC contains micro-clusters of AKAP, PKA, calcineurin and calmodulin in its vicinity. Following channel opening and calcium entry, unique combinations of these enzymes can be activated (Christel and Lee, 2012). Subsequent downstream events include calcium-dependent inactivation, calcium-dependent facilitation, calcium-dependent outward potassium current activation (Veng et al., 2003; Gamelli et al., 2011), release of calcium from internal stores, initiating MAPK signaling cascades, CREB phosphorylation and gene transcription (Deisseroth et al., 1998; Dolmetsch et al., 2001).

Under several circumstances, LTCCs have been shown to be important for long term potentiation (LTP) of synaptic activity. LTP induction in perforant- dentate gyrus synapses is inhibited by chronic administration of an LTCC antagonist (Lashgari et al., 2006). Also, inability to induce LTP in the hippocampal CA1 region of tenascin-C deficient mice is attributable to impaired LTCC function (Morellini et al., 2017). Specifically in olfactory plasticity, blocking LTCCs prevents LTP induction in main olfactory bulb (MOB) slices (Zhang et al., 2010). Naturally, alongside their role in LTP, LTCCs have been deemed essential in numerous forms of learning and memory.

The role of LTCCs in learning and memory has been investigated in many contexts including hippocampus-dependent spatial memory (Ingram et al., 1994; Batuecas et al., 1998; Quevedo et al., 1998), amygdala-dependent fear memory (Bauer et al., 2002; Cain et al., 2002; Suzuki et al., 2004; Davis and Bauer, 2012), prefrontal cortex-dependent working memory (Heng et al., 2011) and others (for review see Berger and Bartsch, 2014). In varied circumstances, administration of LTCC agonists have been shown to improve memory (Jerome et al., 2012) while antagonists can either disrupt memory (Bauer et al., 2002; Cain et al., 2002; Suzuki et al., 2004; Lashgari et al., 2006; Davis and Bauer, 2012), or enhance it depending on the experimental paradigm (Levy et al., 1991; Quevedo et al., 1998; Quartermain et al., 2001). Additionally, CaV_1.2_ and CaV_1.3_ knock out mice have poorer learning abilities (Moosmang et al., 2005; Marschallinger et al., 2015). The importance of LTCCs in protein synthesis-dependent long term memory is well established (Davis and Bauer, 2012; Da Silva et al., 2013). Although most attention has been given to CaV_1.2_ for mediating learning (Moosmang et al., 2005; White et al., 2008), CaV_1.3_ has important roles as well (Marschallinger et al., 2015; Kim et al., 2017).

## LTCC in Olfactory Learning

### Early Odor Preference Learning Model in Rodents

Our laboratory and others have exploited early odor preference learning in neonatal rodents as a model system to understand mechanisms underlying learning and memory, including the role of LTCCs. In this model, the rodent pup is stroked with a paintbrush to mimic a maternal cue while being simultaneously exposed to an odor, leading to an associative memory for that odor which lasts 24 h with one-trial learning (Yuan et al., 2014). Learning occurs within a critical developmental period, between 10 and 12 postnatal days (P10–12), beyond which this association is no longer formed (Sullivan et al., 2000a). NE release from the locus coeruleus (LC) stimulated by the paintbrush stroking provides the unconditioned stimulus (UCS) for this paradigm. Early odor preference learning can also be induced without stroking by directly activating beta-adrenoceptor (βAR) using isoproterenol in the presence of an odor (Sullivan et al., 2000b; Harley et al., 2006; Ghosh et al., 2015).

Another advantage of this model is that it can be manipulated to produce memories of varying lengths, permitting the dissection of both long-term (LTM) and short-term memory (STM). Blocking PKA or protein synthesis produces a STM for 3 h which is not sustainable at 24 h (Grimes et al., 2012). On the other hand, multi-trial, spaced training leads to odor preference memory beyond 24 h (Fontaine et al., 2013), as do the manipulations that either prevent cAMP breakdown (McLean et al., 2005) or block protein phosphatase 2B (Christie-Fougere et al., 2009).

Proper functioning of both the MOB and the anterior piriform cortex (aPC) are critical for this early olfactory preference learning to occur (Sullivan et al., 2000b; Morrison et al., 2013). Although the role of LTCCs in olfactory memory has generally been less explored, it has been shown that LTCCs provide facilitation of this associative learning in both the MOB and the aPC.

### Differential Roles of NMDA Receptors (NMDARs) and LTCCs in Olfactory Learning

LTP and many forms of learning, including early odor preference learning, are initiated by calcium-dependent signaling cascades. It follows that much research has focused on the role of calcium-permeable NMDARs and LTCCs in the context of learning and LTP. While both channels permit learning, their differential contributions to memory formation has been largely overlooked.

**Figure 1:**
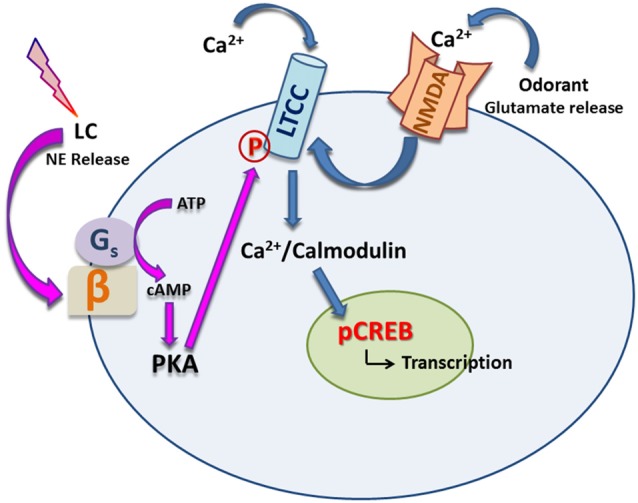
Noradrenergic modulation of L type calcium channels (LTCCs). Exposure to odorant(s) releases glutamate from presynaptic terminals. Together with norepinephrine (NE) release onto the principal cell induced by stroking, postsynaptic NMDA receptors are activated. Subsequent activation of LTCCs allows calcium influx which binds to calmodulin and its kinases (e.g., CaMKIV) and promotes phosphorylation of CREB in the nucleus, leading to transcription and protein synthesis. On the other hand, NE can bind to β adrenoceptors (βARs), which in turn, activates PKA via second messenger cAMP, leading to phosphorylation of LTCCs and further augmentation of calcium influx through them.

Using calcium imaging in aPC slices, Mukherjee and Yuan (2016) demonstrated that LTCC activation is subsequent to NMDAR activation. Behaviorally, it was shown that LTCCs are required for early odor preference LTM but STM can still occur without LTCC activation. LTCC blockade by nimodipine infusion to the aPC prevented LTM but spared STM, while blocking NMDARs with APV prevented both STM and LTM. However, forced activation of LTCCs with BayK 8644 in the presence of APV reversed the loss of both types of memory. This indicates that LTCCs are crucially involved in mediating olfactory LTM in early life and that they can provide the necessary calcium normally supplied by NMDARs to facilitate STM as well. LTM requires PKA-activated CREB signaling as it is both protein synthesis and transcription dependent (Yuan et al., 2003; McLean et al., 2005; Grimes et al., 2011). Owing to their location in the somatic membrane and at the base of the apical dendrite (Mukherjee and Yuan, 2016), LTCCs could be a fit candidate to bridge synaptic excitation and provide necessary calcium to the soma, activating kinases which subsequently phosphorylate CREB to initiate transcription in the nucleus and ultimately protein synthesis. Activation of LTCCs following NMDAR activation in the aPC is consistent with this hypothesis (see Figure 1).

Calcium-dependent AMPAR insertion is critical for the formation of both short and long olfactory preference memories, and in line with behavioral results, blocking NMDARs with APV in the aPC prevented the increase in synaptic expression of AMPARs required for STM (Mukherjee and Yuan, 2016). The increase in synaptic expression of AMPARs was rescued when APV was co-infused with BayK 8644, suggesting that the calcium influx from LTCCs alone is sufficient to mediate AMPAR trafficking to the synapse. Although able to induce STM, behaviorally co-infusion of BayK 8644 and APV compromised stimulus specificity of the learning. Animals showed preference to the conditioned odor (peppermint) against a control odor (vanillin), however, failed to form preference to peppermint against a similar odor mixture (peppermint + vanillin). This suggests that stimulation of LTCCs in the absence of NMDAR activation results in the trafficking of AMPARs into a broader range of synapses and thereby a loss of stimulus specificity occurs for two similar odors (Mukherjee and Yuan, 2016).

### LTCC Modulation by NE during Olfactory Learning

NE is an important neuromodulator that can engage α1, α2, or βARs and initiate G_q_, G_i_, or G_s_-mediated downstream cascades, respectively. The differential effects of NE through these G-protein coupled ARs has been established to be critical for several olfactory learning paradigms (Sullivan et al., 2000b; Doucette et al., 2007; Lethbridge et al., 2012; Shakhawat et al., 2012, 2015; Morrison et al., 2013). As NE is released from the LC of pups in response to stroking, serving as the UCS in early odor preference learning, the effect of NE on modulating LTCC function in olfactory memory context is studied in this paradigm.

In the MOB blocking LTCCs by nimodipine infusion prevents early odor preference LTM. However, activation of LTCCs through BayK 8644 is not sufficient to rescue APV induced LTM loss; βAR activation is also required to rescue LTM (Jerome et al., 2012). LTCCs are present on both glutamatergic MCs and GABAergic periglomerular cells (PGCs) of the MOB (Jerome et al., 2012) and it has been shown that activation of PGC LTCCs triggers GABA release (Murphy et al., 2005). It could be that βAR activation suppresses PGCs (Yuan, 2009) and counteracts LTCC activation on PGCs, leading to MC excitation and ideal calcium influx through MC LTCCs. Additionally, as shown in the aPC (see Figure 1), NE can upregulate LTCC-mediated calcium influx in pyramidal neurons through βAR-mediated PKA-dependent pathways and thereby promote learning (Ghosh et al., 2017). Accordingly, PKA-mediated direct upregulation of MC LTCC activity could underlie early odor preference learning in the MOB. These findings suggest that LTCC activity serves as an important postsynaptic correlate of NE’s facilitating action on olfactory memory. In line with these reports, NE-mediated LTP has been found to be dependent on LTCC activity (Zhang et al., 2010).

The effect of βAR activity on LTCC function and the role of PKA in exerting that effect have been explored in neurons. βAR activation inhibits calcium-dependent inactivation of high voltage gated calcium channels including LTCCs and this mechanism likely involves PKA and A-Kinase anchor protein (AKAP; Rankovic et al., 2011). Additionally, a highly localized β_2_AR-PKA dependent increase in LTCC activation and subsequent calcium influx is observed in spines of CA1 pyramidal neurons (Hoogland and Saggau, 2004). A recent report highlights the unique importance of serine 1928 of CaV_1.2_ in relation to PKA-mediated phosphorylation (Qian et al., 2017). Phosphorylation of serine 1928 is required in hippocampal neurons for LTCC activity augmentation by PKA. A long term potentiating effect in response to prolonged theta stimulation required an increase in CaV_1.2_ activity following modification of serine 1928 by the β_2_AR-cAMP-PKA pathway, but not β_1_AR. Interestingly, β_1_ and β_2_ may have opposing actions on LTCCs as reported in adrenal chromaffin cells where β_1_-mediated enhancement of LTCC function is dependent upon G_s_ and PKA but β_2_-mediated decrease in LTCC function involves G_i_ proteins (Cesetti et al., 2003). Such diversification in molecular targets and mechanisms may be relevant for LTCCs in neurons of the olfactory system too. In aPC pyramidal neurons, a βAR agonist isoproterenol exerts augmentation of LTCC activity in P7–10 mouse pups, but not in older pups beyond 2 weeks of age. This effect is PKA-dependent and crucial for early odor preference learning (Ghosh et al., 2017). However, whether this effect is mediated by β_1_ or β_2_ is still undetermined. It will be important to understand how different βARs contribute throughout the lifespan and whether or not a dichotomy lies in their effect to form olfactory memory.

## Roles of LTCC in Adult Learning and Aging

The most recent work by Ghosh et al. (2017) explored the developmental changes of LTCCs in aPC pyramidal neurons by comparing whole-cell calcium current recordings before and after the critical period for early odor preference learning. The proportion of LTCC-mediated calcium influx with respect to the whole-cell calcium current decreases beyond the critical period, up to the weaning age. Blockade of LTCCs by nifedipine infusion in the aPC prevents early odor preference learning during the critical period. Therefore LTCCs are situated as a key factor for early life odor preference learning in rodent pups.

Although early life developmental changes in the aPC were followed by a decreased proportion of LTCC-mediated current in older pups (Ghosh et al., 2017), in adult life, LTCC-mediated calcium current increases with age. This apparent contradiction could be explained by relatively higher expression of non-LTCCs in P14–20 pups. While this idea remains to be tested, differential calcium channel expression in early and later postnatal life cannot be ruled out. An enhancement of LTCC function and postsynaptic PKA signaling has been reported in prefrontal cortex from P25 through P80, likely to be associated with improved working memory and decision making during early adulthood development (Heng et al., 2011), highlighting the age-dependent balance of LTCC-mediated current. However, a large body of evidence exists supporting the idea of increased calcium influx being correlated to advanced aging (Khachaturian, 1987; Landfield, 1987) and causal to the impairment of memory. LTCCs hold an important position in this calcium hypothesis of aging. Increased calcium influx through the LTCC has been reported in aged CA1 pyramidal neurons, contributing to cognitive decline and memory deficits (Moyer and Disterhoft, 1994; Thibault and Landfield, 1996). It has been proposed that instead of a global increase in the number of LTCCs, increased expression and density on the cell membrane might underlie the increase in age dependent LTCC calcium entry (Núñez-Santana et al., 2014).

If LTCC is crucial for protein synthesis dependent long term memory and LTCC current increases in aged animals, why is there a deficit in learning abilities rather than an enhancement of it? Reports suggest that a reduction in neuronal intrinsic excitability may explain this fallacy. Calcium dependent outward potassium current gives rise to an after hyperpolarization (AHP) current and its slow component has LTCC dependance, especially on CaV_1.3_ (Veng et al., 2003). Both AHP magnitude and its dependance on LTCC increase with age in CA1 neurons, making them less likely to generate trains of action potentials, thus leading to a decrease in intrinsic excitability and possibly a decreased ability for the animal to learn (Power et al., 2002; Oh et al., 2016). Olfactory learning related changes in NE-modulation of AHPs have been proposed to keep the post-learning hyperexcitability of the network in balance (Brosh et al., 2006). Whether this change is dependent upon LTCCs remains an open question. Also in senile subjects a higher tendency to form LTD increases with enhanced LTCC function (Norris et al., 1998). Together these findings suggest that an optimum level of LTCC function is required to keep the balance between facilitation and impairment of learning.

Although it seems clear that NE and LTCCs are important players in olfactory learning and memory, several questions remain unanswered. Do different ARs modulate LTCCs uniquely in the olfactory system? Does LTCC activity change accordingly with age? Is LTCC-dependent AHP an important player in olfactory learning? If so, how does NE modulate LTCC-dependent AHP? Does the age dependent dichotomy in β-adrenergic modulation of LTCC function in early life play a similar role in senescent plasticity as well? Future studies should be directed towards these questions to bring about a clearer understanding of the diverse roles provided by the crucial players in learning and memory.

## Conclusion

In the olfactory system, a concerted effort by NE, NMDARs and LTCCs in both the MOB (Jerome et al., 2012) and aPC (Mukherjee and Yuan, 2016; Ghosh et al., 2017) can produce the phenomenon of early odor preference learning, a memory which lasts only until approximately P10–12, corresponding to a sensitive developmental period in rodents. LTCCs may aid in the formation of LTM by promoting nuclear transcription, protein synthesis and sustained AMPAR synaptic expression in the aPC (Mukherjee and Yuan, 2016). In the same type of neurons, the proportion of LTCC-mediated calcium decreases following the sensitive period compared to older pups up to the weaning age. Of great interest, LTCCs lose the βAR-mediated PKA modulation beyond the critical period (Ghosh et al., 2017). This might be one of the key mechanisms to explain why rodent pups lose their ability to form olfactory association with maternal cues past the critical period. The unique roles of LTCCs in the early developmental period, adulthood and aging animals appear distinct and require further investigations.

## Author Contributions

AG, QY and XC developed the idea. AG did the literature review. AG and SJC wrote the manuscript. Further editing was done by AG, SJC, XC and QY.

## Conflict of Interest Statement

The authors declare that the research was conducted in the absence of any commercial or financial relationships that could be construed as a potential conflict of interest.
